# A Doctor's Visit to U.S.S.R.
*The Long Fox Memorial Lecture, delivered on 16th October, 1956.


**Published:** 1957-01

**Authors:** A. M. Maclachlan


### A DOCTOR'S VISIT TO U.S.S.R.*

BY

A. M. MACLACHLAN, M.B., CH.B.

I look upon it as a great honour to be invited to give the Long Fox Memorial Lecture for 1956, the more especially as my twenty-five years in medicine have been spent in the field of general practice, away from the University classroom, the laboratory, and the hospital bed. General Practitioners are not used to giving lectures; when they do they are wise if they confine themselves to the bird's-eye rather than the worm's-eye view and in the language of the Authorized Version "speak the things that they do know and testify the things that they have seen".

My trepidation in appearing in the role of lecturer is somewhat sedated by the terms of the appointment—that the lecture is expected to be a popular rather than strictly scientific one, and that although the subject-matter must be about some aspect of medicine it should appeal to a non-medical as well as a medical audience.

In July of this year I had the good fortune to be a member of the civic delegation from Bristol which visited Saratov in Russia, and as I took the opportunity of finding out something of the progress of medicine in Russia in general and Saratov in particular and visited several of their hospitals and clinics, I have taken as my subject tonight "A Doctor's Visit to U.S.S.R."

It may be objected that one can see little cf another country in a fortnight—especially in the field of medicine. That is a fair criticism. But I think it also fair to say that the seeing, however brief, and the impressions, however personal, may not be without value if one goes to Russia with open mind and with open eyes. Yet it is also true to say that personal contact with the Soviet Union is still charged with considerable emotion on both sides, and it is sometimes difficult to separate a personal reaction from an impartial assessment of conditions and work in U.S.S.R. One must also resist the human temptation to be an authority on such a subject after so brief a visit and simply trade on the greater ignorance of one's audience just because they have never been there.

There is also the language difficulty. The interpreters do their best but one is not always sure that the question has been understood or that it means the same thing in Russian as it does in English. We were more fortunate than some other delegations in that before accepting the invitation to visit Saratov the Town Clerk insisted on having an interpreter from the British Embassy. This I am sure saved us from many a pitfall. On many occasions she saved us from getting a completely false impression from an answer to a question just because the Russian interpreter wanted to put a polite gloss on either question or answer.

### ADMINISTRATION

To understand the structure of the medical services in Russia one must remember that the country is a federated state of sixteen sovereign republics each with its own constitution. But this sovereignty is nevertheless restricted so as to create a uniform system in a planned society. Within the different republics further divisions are made consisting of the region, sparsley populated region, and district. Again, the large towns are divided into districts like our metropolitan boroughs. Each of these units has its own Soviet or local Executive which directs the activities of administration, local economic and cultural affairs and draws up a local budget.

Each of the Union's autonomous republics has its own Ministry of Health which controls the whole of the Health Services within that republic—preventive, diagnostic,

* The Long Fox Memorial Lecture, delivered on 16th October, 1956.

curative, medical education and research. In the region you have the same set up wi a Director of Health responsible to the Director of Health of the Republic, and course the Director of Health of the Republic in turn is responsible to the Ministry Health of the U.S.S.R. in Moscow, which works out policy and directs and coordina the work of the sixteen republics. Even in the smallest unit—the district—which m be but a limited area in a city the set up is the same; and it is laid down by law th each district must have its own Health Department. At the head is the Director Public Health for the district with over-riding powers, and responsible for all hea services in his district including hospitals, polyclinics, sanitation, midwifery servic and control of epidemics. It will be seen that in the U.S.S.R. there is nothing like three great divisions in the National Health Service—Hospital Services, Lo Authority Services, and General Practitioner Services, each with its own controlli and directing administration. Nor is there the same divorce between administrati work and clinical work. The Director of Health of the district is not only responsib for the smooth running of the administration in his area but he does clinical work well and takes his turn at the polyclinic.

From what I was told emphasis in the Soviet Union seems to be on (1) Preventi Medicine, (2) Maternal care and child health, and (3) Industrial medicine.

### (1) PREVENTIVE MEDICINE

By this they mean B.C.G. vaccination, routine examination of workers, diphtheri inoculation, the wide use of antibiotics in prophylaxis, with less emphasis on sanitati and clean food than in this country. Since nearly all babies are born in hospital, B.C. is given compulsorily within two or three days of birth. Administration of the vacci is repeated at 7, 12, 15 and 18 years of age. Incidentally the medical professio U.S.S.R. is very proud of the spectacular fall in their death-rate from tubercul during the last decade. They do not seem to be aware that this is something which happening throughout the civilized world and has nothing to do with the superio of Soviet medicine over that of other countries in Western Europe. In many ways th still show all the arrogance of their insularity.

### (2) MATERNAL CARE AND CHILD HEALTH

They make a great deal of this in U.S.S.R. The child is all-important and protection starts in the ante-natal clinic. In the first weeks of life he attends paedriatic clinic, and later if his mother is at work he is very well looked after in crèche attached to the factory. School children are examined regularly until the of 17. Large holiday camps and health resorts are available for the young. We vis one where on the wooded slopes of the hills above the town 1,300 children w spending about four or five weeks in camp during the school vacation.

### (3) INDUSTRIAL DISEASES AND THEIR TREATMENT AND THE IMPROVEMENT OF INDUSTR CONDITIONS

I was struck by the emphasis on physiotherapy and hydrotherapy, especially the polyclinic attached to the factory, and the importance given to rehabilitation the need to get the worker back to his job at the earliest possible moment. We visi one light engineering factory which employs 8,500 workers, 60 per cent. of th being women. The large polyclinic, which is part of the factory organization, a staff of twenty-five doctors who work in three eight-hour shifts. The first half of shift the doctor spends in routine examination of workers or in treatment of mi illnesses; the second half of the shift he spends in the workshop examining wor conditions—working out schemes for the improvememt of conditions of work incidentally, but most important in the U.S.S.R., for the speeding up of product Many of you may think this strange work for a doctor but you must remember th Soviet Russia a doctor prides himself on being a member of his trade union. "Statutes of Trade Unions of U.S.S.R." state that trade unions exist to "orga competition for the maximum growth of productivity of labour" and even a doctor is proud to make his contribution towards this end. Trade Unions in Russia do not exist to press wage claims on behalf of their members but to "develop competition for the fulfilment and over-fulfilment of production targets".

We were told that due to the improvement in industrial hygiene, illness and industrial accidents have decreased by three times in recent years. But it must be remembered that Soviet Union industrial standards are much below ours and we saw conditions in a ball-bearing factory which would have been condemned out of hand by our Factory Acts of the nineteenth let alone those of the twentieth century.

### SOCIAL INSURANCE

The Soviet Union has a Ministry of Social Insurance responsible for the care of workers and their families who are unable to work for any reason. Pensions are provided for three categories (1) Those incapacitated by disease or injury: (2) Old age pensioners: (3) War pensioners. There are a number of differences from social insurance in our country, for instance disablement pensions are non-contributory; old age pensions are not fixed but range from 50 to 60 per cent. of wages, the pension being computed on the basis of the average earnings over the last five years. Another difference is, if a man is killed at work his widow gets a pension only if she is unable to work, while the children get a pension apart altogether from the mother's earnings.

### THE CARE OF THE AGED

I was speaking just now about the importance of ante-natal care and the care of children in the Soviet Union. In direct contrast to this is their attitude to the old. Pension schemes are said to operate after sixty years of age but we saw many who were over sixty or who must have been prematurely old doing work which would only be undertaken by able-bodied men in this country. We saw old women sweeping the street at four o'clock in the morning (most of this type of work is done by elderly women); we also saw younger women working at furnaces, driving cranes, working as plate-layers on the railway, and working as brick-layers alongside men.

On our way to Russia we spent a day at Helsinki, and on our return a day at Copenhagen. In both cities we were given a civic luncheon and afterwards taken on a sight-seeing tour of the city. In both countries we found them very proud of the provision they were making for the care of the elderly, whether in the provision of special blocks of flats or special homes or Geriatric Hospitals. We saw nothing of this in Russia. Saratov which has a population of 600,000 makes no special provision for the elderly or the chronic sick and does not have a single Geriatric Hospital. When I asked about this I was told that there were now several Geriatric Hospitals in U.S.S.R. and that Saratov hoped to build one during the sixth five-year plan, that is during the next five years. One learned, however, that when in Russia they say they hope to do a certain thing, it indicates it is not on the priority list and no special plans have so far been made.

### MEDICAL EDUCATION

After the revolution of 1917 the need for reorganizing medical training was acute and great efforts were made to increase the quantity and the quality of medical personnel. At first the entrance requirements were exceedingly low, in some cases a mere formality, and as a result, there grew up a generation of poorly equipped medical practitioners. At this time the training was the responsibility of the Ministry of Education, but in 1930 training was made the responsibility of the Ministry of Health. Under the new Ministry the aim was to produce three types of physician.

(1) Practitioners for general medical, surgical and prophylactic work.
(2) Public Health physicians.
(3) Specialists for protection of mother and child.

Medical Schools therefore divided into three corresponding faculties:

(1) Faculty of General Medicine (4-year course)

(2) Faculty of Hygiene ($3\frac{1}{2}$-year course)

(3) Faculty for Protection of Mother and Child (4-year course)

This new system, however, proved unsatisfactory and later in the 1930's they reverte[illegible] to the five-year curriculum common to medical schools in this country. In 1945 th[illegible] curriculum was extended to six years, the final year to be used for practical work i[illegible] hospital, polyclinics and medical centres. Before the Second World War the gre[illegible] problem was the inadequate pre-medical education of students. The standard of gener[illegible] education in Russia is (in theory at least) of ten grades from 6 to 16 years of age o[illegible] 7 to 17 years of age. It was found, however, that many of the students wishing t[illegible] start a medical career had only completed seven grades instead of ten and even tod[illegible] such is the shortage of schools and teachers that many schools work in a two-shi[illegible] system, half the children attending in the morning and the other half in the afternoo[illegible] The Russians got over this difficulty of inadequate pre-medical education by admitti[illegible] students to a "secondary Medical School" from which they graduated as "feldshe[illegible] nurse or midwife after a three-year course. They then practised for three years, [illegible] feldsher, nurse or midwife after which they applied for admission to a medical scho[illegible] Other students below entrance standard went to work in a factory in the daytime and [illegible] the same time attended special night schools where they studied a foreign langua[illegible] mathematics, physics, chemistry and political science so that in three years time th[illegible] might meet the entrance requirements of the medical schools.

As has been indicated the entrance examination to the Medical School consists [illegible] a foreign language, mathematics, chemistry, physics and political science. Germ[illegible] used to be the most popular language but now by far the largest number of studen[illegible] take English. This we were told is the case, not only in the medical but in all the oth[illegible] faculties as well. There is a great drive to learn English. The extraordinary thing [illegible] that of all the doctors whom we met in Saratov from the Director down to the doctor [illegible] the clinic we did not meet one who seemed to know any English. A member of th[illegible] British Embassy staff in Moscow had to go to the principal Moscow hospital for [illegible] emergency operation the other month and an interpreter from the Embassy had to [illegible] with him as not one of the surgeons or members or the staff of the hospital kne[illegible] English. One wonders how much of the required examination standard is like so ma[illegible] things in Russia "on paper only".

On the general question of education it has to be remembered that before the revol[illegible] tion of 1917 the standard of literacy in Russia was very low and more and more the[illegible] seems to be a desire to follow what is best in the education system of countries l[illegible] England. Perhaps the most startling innovation is that proposed by Kruschev in h[illegible] speech to the Twentieth Party Congress in February 1956. I quote from the official co[illegible] of the speech which I purchased in Moscow. "It is now expedient," said Krusche[illegible] "to start building Boarding Schools. Children should be enrolled in these boardi[illegible] schools at the request of their parents. They will live in the schools and their pare[illegible] will visit them on holidays and during vacations. Children whose parents do not ea[illegible] much should be fully maintained by the State. Parents with higher earnings should p[illegible] part of the cost of the education of their children. Finally some parents could co[illegible] fully the outlay made by the State on the education of their children in the boardi[illegible] school."

I had the opportunity of visiting the Medical School in Saratov which is called th[illegible] Medical Institute. It is not part of the University buildings, and I got the impressio[illegible] that it was not looked upon as part of the University. It is said to be one of the olde[illegible] Medical Institutes in the country dating back to 1909. Four hundred and fifty stude[illegible] graduated from the Institute this year. This is a staggering number when you think [illegible] Saratov as a city just somewhat bigger than Bristol. But it gives an idea of the enormo[illegible] technical drive and the tremendous educational expansion which is taking place [illegible] Russia. The figures given for the increase in the number of doctors are interesti[illegible]

At the Revolution there were 20,000 doctors in the whole of Russia.

| | | |
|---|---|---|
| In 1928 | .. .. .. | 63,000 |
| 1932 | .. .. .. | 76,000 |
| 1938 | .. .. .. | 112,000 |
| 1941 | .. .. .. | 130,000 |
| 1954 | .. .. .. | 300,000 |

'he curriculum is much like the medical curriculum in England with some striking xceptions. For instance, 246 hours are given to clinical surgery but 250 hours to the udy of the principles of Marxism-Leninism. When the student goes on to his clinical udies at the bedside, he attends the Institute in the morning for theoretical training nd the Hospital for clinical teaching in the afternoon. Sixty per cent. or more of the udents are women. When a student graduates he has no free choice as to what work e will take up or where he will go. In general young graduates are sent for several ears' practice (usually three) to the rural districts and the sparsely populated areas. t the moment there is a great drive to open up virgin land for agriculture and an ttempt to develop vast areas in Siberia and put them on an economic basis. There are hemes for afforestation and wheat growing, with hydro-electric power schemes on a cale never attempted before. Thousands of young technicians having just passed their nal examinations are being drafted into these areas. More and more collective farms are pringing up, and they all need doctors. It is in such conditions that the young medical raduate learns to become a general physician, to depend on his own resources, to evelop self-confidence—in a word, to learn the hard way.

After three years he may return to the city and apply for a position in the polyclinic r enter hospital and specialize, or if he has been a particularly brilliant student, with pecial aptitude for scientific work, he may on the recommendation of his teacher ecome a research worker. What are doctors paid? The young doctor is paid 600 to ,000 roubles per month. To assess how much that is worth it is necessary to compare with the wages of some other workers. A washer-up in a canteen gets 400 roubles and fitter may get 1,200 roubles. A cook in a large canteen gets 2,000 roubles, a manager of canteen 3,000 roubles, a reporter 3,000 roubles. On the other hand a higher medical ualification may mean 1,700 roubles a month, a professorship 5,000 roubles, and at the op of the tree is a Director of Research with a maximum of 11,000 roubles a month. t will be realized that whatever kind of society they may have in U.S.S.R. it is by no neans a class-less society. It would appear that although all do equal work some salaries re more equal than others. It follows that there are those whose standard of living is question of making ends meet; there are others who can afford to buy a house (and it is ow possible to buy a house in U.S.S.R.) or a motor-car, or to invest surplus money in Government stock at 3 per cent.

### HOSPITALS

I will now try to describe what I saw of clinical medicine and my impressions of the ifferent hospitals I was allowed to visit when in Saratov. Saratov is a large industrial ity 500 miles south-east of Moscow, lying on the right bank of the Volga, north of talingrad. The town was founded early in the sixteenth century on the left bank of the olga; later it became a small fishing centre and was transferred to the right bank of he river. It is surrounded by hills from one of which (the Sara Tov or yellow mountain) takes its name. Its geographical position and its natural resources settled the town's conomic position in the country, and by the middle of the seventeeth century it had ecome a trading centre. In the eighteenth century it became a regional town but its ndustrial resources were not developed till after the revolution of 1917. The increase n the industrial side in the past forty years has been phenomenal. During the last ve-year plan (the fifth) it more than doubled its industrial output and today it is one f the largest industrial centres on the Volga. The gas supply of Moscow comes from aratov, which is rich in natural gas. A 500-mile-long pipe-line carries the gas from aratov to Moscow.

The largest General Hospital has 630 beds—270 surgical beds, 130 medical beds 230 gynaecological beds. Each department is housed in a separate building, the th buildings being about 50 yards apart. Structurally the buildings are poor and fa outside in a bad state of repair. Both inside and outside they resemble our Law hospitals of 25 years ago rather than a modern general hospital attached medical school. The beds are very low and of poor quality. I had a look at som the temperature charts and got the impression that charting was perfunctory inadequate. They do not seem to have any difficulty in obtaining nursing staff, th being far more nurses than in the wards of a hospital in England. I did not see male nurses. The nurses do not wear uniform but a white gown over indoor clothe could not obtain much information about the standard of their training althoug understand the course for nurses and midwives is three years. Nor could I find how efficient was the nursing. In this connexion, however, it must be rememb that there is no nursing tradition in Russia as in England, and up to 1917 there no profession of nursing at all. I was not allowed to see any of their sluices or lavat —although I asked to. Lavatories are things they do not want you to see and on occasion when we were permitted to see two workers' houses all the rooms were to inspection save the lavatory which was locked and the key removed. The ward this hospital were small having four to eight beds and the ventilation was very indeed. This ventilation problem was one which we observed in practically building in Saratov, and everywhere was the Russian smell. The vitiated air ma due to the use of double doors and windows because of the extreme cold in the win on the other hand in Helsinki all the windows and doors are of the double variety ventilation is excellent by English standards. Equipment and methods of treatm presented strange contrasts. Dry cupping, and leeches in the treatment of heart failure on the one hand, and lavish equipment for physiotherapy and a wide ra of antibiotics on the other. Electrical equipment seemed to be all over the place. gadget demonstrated to me was in the form of a cage about 6 ft. high, inside wh the patient sits on a wooden stool and round him is created an electromagnetic fi I was told it was used in the treatment of melancholia, migraine and tic doulour

I gathered that they used most of the antibiotics in use in this country but informed that a new antibiotic called Biomycin had recently been developed by So research workers. I was given a sample with the requisite literature which I subm to Messrs. May and Baker on my return to England. May and Baker tell me th appears to be identical with or closely related to Chlortetracycline. Russian doctor not seem to worry about acquired resistance to antibiotics and use them on a far gre scale in prophylaxis than we do. A special line of their own is to use penicillin or antibiotics by inhalation in the prophylactic treatment of upper respiratory dis I was told that this prophylactic treatment is used at the beginning of any infectio the influenzal type. Workers in the factory are encouraged to attend for this treatm at the onset of an acute rhinitis or if they have a sore throat. They use the sulpha d in the same way. P. K. Bulatov in his book "*Modern Methods of Treating Bron Asthma*" which I purchased in Moscow says "In the case of 32 patients affected chronic non-specific pneumonia when prophylactic treatment with one grain sulphanilamide two or three times a day was recommended attacks of bronchial ast which generally set in after the inhalation of various odours (smoke and dust, mois cold air, etc.) were prevented for a period of two to three months." They claim fol ing the teachings of Pavlov that sulpha drugs act on the central nervous system have a disensitizing effect. Bulatov claims that sulphonamides exercise a desensiti as well as a bacterostatic influence. They also treat bronchial asthma with intraven injections of a 25 per cent. solution of novocaine, sometimes giving intra-trac injections of novocaine as well. Another method in the treatment of bronchial ast is by means of what they call bilateral jugular vago-sympathetic and intra-cutan blocks.

In the surgical unit of the General Hospital they have one X-ray room and one X

ble. I saw only one operating theatre but it had three operating tables and it appears be customary for three surgeons to be working in the theatre at the same time. The om for sterilizing equipment was not on the same floor as the operating theatre. The ash basins which the surgeon used for scrubbing up had the ordinary domestic tap ndles. An orderly turns the taps off and on as required.

They do not make the same use of general anaesthesia as in this country but appear have reached a high standard in the perfection of local and nerve block anaesthesia.

The Hospital kitchens and methods of feeding were unimpressive. The food is rried to the wards in standard, old fashioned bucket shaped utensils and there was method of keeping food hot in its transit from the kitchen to the ward.

I asked about blood transfusion units and was told they have a blood donor service d blood banks as in England. The Russians have apparently perfected technically e use of cadaver blood. They say that in twenty-five years they have had no compli- tions and no deaths by the use of this method. The procedure is very simple. The in is disinfected with iodine, two cannulas are introduced into the internal jugular ins and the corpse is brought into such a position that the blood flows freely. About o or three litres of blood are extracted in this way and can be preserved in an ice x for 28 days.

I tried to get some information about mental hospitals. Russians doctors were shocked hen I mentioned the percentage of beds given over to mental illness in this country. hey assured me that the 100 beds they had for mental cases in Saratov were more than ough and that in the Soviet Union there is no such thing as anxiety neurosis. Since ey still think that England today is the England of Charles Dickens, that e have widespread unemployment, that children go barefooted to school, they are t surprised that anxiety neurosis is fairly common in England. When I tried to rrect one Russian's false impressions of England she told me she knew what she as talking about. Had she not read *Oliver Twist* and *Mrs. Warren's Profession*.

Two other hospitals were visited in Saratov—the Orthopaedic Hospital and the incipal Maternity Hospital. The Orthopaedic Hospital was opened in 1945 to commodate and treat men returning from war with severe damage to limb. Before ing taken over as an Orthopaedic Hospital it had been a school. The building was erefore an adapted one and not at all suitable for Hospital purposes. During the ar about one-third of the school buildings in Saratov had to be used for hospital rposes. Cases demonstrated included neurological surgery, plastic surgery, and thopaedic surgery. Several cases of rickets were being corrected by orthopaedic rgery. They seemed surprised when I told them we did not now see rickets in ngland.

The principal Maternity Hospital has 80 beds with 2,000 cases per year. There is domiciliary midwifery, all confinements taking place in Hospital. The appalling using conditions may have a great deal to do with this. We were not able to obtain ures about housing, and overcrowding is not a subject the Russians are prepared discuss, but it was obvious to us that in some parts of this great provincial city ople were housed like animals rather than human beings. Moscow which has a gher standard than other cities in U.S.S.R. has stepped up new buildings so that w they can offer one room per family.

An antenatal clinic is attached to the Maternity Hospital, and there being no miciliary midwifery there is no district midwifery service. Health visitors, however, sit the homes when the mothers go home and encourage them to keep in touch with e clinic. Altogether there are six maternity hospitals in Saratov with a total of 500 ds. The still-birth rate, I was told, was 1 per cent., forceps cases 2 per cent., d Caesarian section 1 per cent. They do medical but not surgical induction. When I ked about this and sketched a Drew Smythe Catheter they had not heard of it. xaemia they say is rare and the eclampsia rate 0.008 per cent. Sedatives are seldom ed. They do not use either triline or pethedine and have no use at all for general aesthesia. I visited the labour ward. There were three beds in line about two feet apart and a woman in an advanced state of labour on each. The Pavlov meth[illegible] painless labour was demonstrated. It appears to be part hypnotism and part [illegible] taught relaxation on a woman well conditioned beforehand. The instrument[illegible] was a shock. A table about three feet square covered with oil cloth but no sterile [illegible] The equipment consisted of a small sterilizer with a few instruments, a do[illegible] saucepan containing sterile water and one kidney dish. The attendants were[illegible] wearing sterile gowns or gloves. There, as in the other Hospitals which I visi[illegible] formed the opinion that Russian Hospital standards of cleanliness and sterili[illegible] are very much below ours.

### POLYCLINICS

There is no General Practice in Soviet Russia as we understand it. An indiv[illegible] does not have someone whom he looks upon as his doctor, nor is there such a thin[illegible] family doctor—a practitioner who looks after father, mother and children as well[illegible] Russian citizen sees himself much more in relation to a group than we do, the [illegible] being the factory, the school or a block of flats. In this country a man will say "I'[illegible] well, I must go to my doctor"; a man in Russia will say, "I must go to the polycl[illegible] It may be that father attends the polyclinic at the factory, mother, perhaps pregna[illegible] under the care of the clinic obstetrician, a sick child at home is being visited [illegible] paediatrician while another young adult in the family may attend the district polyc[illegible] There appears to be much more specialization outside the hospital than in Eng[illegible] Each polyclinic has a wide variety of specialists though specialization does not [illegible] sarily confer consultant status.

I visited the Central Polyclinic in Saratov which again is an old building in a [illegible] state of repair outside. Inside, a wide dirty iron staircase leads to the consulting r[illegible] on the first floor. The woodwork is painted a dark-brown workhouse-looking co[illegible] floors are dirty and there is a general absence of ordinary cleanliness let alone sp[illegible] polish. Admittedly many of these places are old but could without much expen[illegible] of money be made bright and cheerful and at least they could give them a ge[illegible] feeling of cleanliness if they but tried.

The polyclinic is in many ways like the out-patient department of one o[illegible] hospitals. They have X-ray rooms, a pathological laboratory, a large departme[illegible] physiotherapy, a whole basement given over to hydrotherapy and a small ope[illegible] theatre for minor surgery. Emphasis I felt was on treatment rather than diagno[illegible] treatment primarily aimed at getting men and women fit for industry again[illegible] doctor attitude struck me as having something of the football trainer about it[illegible] must get this man fit in the least possible time". I have it on reliable authority[illegible] Moscow now has a polyclinic for private patients where you may pay for treat[illegible] The advantages are said to be saving of time for the patient to whom time is impo[illegible] and a more individual approach on the part of the doctor.

There is a special Central Dental Polyclinic in Saratov, which is the training [illegible] for dental students. Most of the students are women. We saw quite a lot of dental [illegible] going on, extractions, fillings, fitting of dentures and making dental bridges. [illegible] make very few dentures and those we did see were not very attractive. Like the [illegible] sold in the shops the quality and finish were poor. There was no attempt made to [illegible] teeth or make the dentures look like natural teeth. Dental bridges are construc[illegible] stainless steel and when you get a patient with a mouthful of teeth made of sta[illegible] steel the result in our eyes is grotesque. But tastes differ: I was told the Ru[illegible] think steel teeth an aid to beauty especially if you have added a gold filling or t[illegible] the remainder of your own teeth which hold the steel bridge together.

No general anaesthetics are used for major dental surgery. Resection is done [illegible] 2 per cent. novocaine. No more than two teeth are extracted at a time and extensi[illegible] is made of antibiotics both prophylactically and locally for infected cavities and th[illegible]

These were some of the things I saw in Saratov. But you cannot learn a great d[illegible] a fortnight especially if you have to share the services of an interpreter with others.

rgent questions which were asked remained unanswered. I was told that cholera, so fe in the old days in that part of Russia, is now so rare that students go through the hole of their training without seeing a case. There are only sporadic outbreaks of phoid now and no malaria. They have only a few cases of diphtheria per year and no eaths. There are occasional outbreaks of poliomyelitis but practically never with aralysis, but so far they have not been able to start inoculations although a start was ade in Moscow this year.

Yet there were the many questions unanswered. The birth-rate, the death-rate, the fant mortality rate: measures to ensure a pure water supply. (The tap water in my edroom was a nice rusty colour due, according to one of the doctors, to work going on ear our hotel, but due according to a member of the local Soviet to the amount of aluable iron in the water supply of Saratov.) Do you have a system of chlorination or lteration? What are your methods of sewage disposal? These were a few of the uestions asked and asked again but in the end they remained unanswered. Medical atistics are not a strong point in U.S.S.R. and I believe it was only about two years go that the first medical statistics for the country as a whole were issued from Moscow. doubt if there are any local statistics for a city like Saratov. If they exist at all they re probably mostly guesswork.

SOCIAL PROBLEMS

(1) *Housing.* In 1917 Russia had not in many ways changed for generations. It was rgely the Russia of the mid-nineteenth century novelist with 80 per cent. of the opulation illiterate peasants, either the children or grandchildren of serfs, engaged in griculture of a very primitive kind and living for the most part in the thousands of easant villages of the country. Today nearly half the population works in towns. The opulation of Saratov has increased by 100,000 since the war. No wonder they have a ousing problem. So far as we could see such has been the concentration on heavy ndustry that only in recent years has any attempt been made to meet housing needs. Ve saw the two extremes—the modern blocks of flats begun no more than a few years go and the tumbledown wooden houses with their tiny fret-work windows and orrugated iron roofs without plumbing or modern sanitation which must have been uilt before the revolution. Even the modern houses are overcrowded by our standards. )ne we saw had a single bedroom for father, mother, grandmother and one child f about four years old.

(2) *Food.* Another problem is food. There is just not enough food in the country— ven now—to go round and during the hard times anyone who was high minded enough o insist on living on his rations simply died. So far as I could judge from what I saw in aratov, Russia has a black market in food run with the connivance of the State. In aratov the market is housed in a building provided by the State and the various stalls re rented from the local authority. In the market you can buy meat, fish, fowl, veget- bles, fruit, bread, all at prices ranging from 50 per cent. to 100 per cent. above the ixed price in the State shops. Why then not stick to the State shops? Because there is ot enough to go round. The market is really a black market. Where does the food ome from? Well, collective farms must produce a certain amount of food as laid down y the local authority, but by working hard and working longer hours they produce a ittle more than their quota which they can then take to the market and sell at whatever rice the customer is prepared to pay. Then there are workers on the State farms who ave to give $x$ hours of work to the State. But when they do $x+1$ hours the produce rom the extra hour is their own which can be sold in the market for what it will fetch.

(3) *Shortages and Crime.* The shortage of consumer goods has led to other social roblems—problems with which we were not unfamiliar ourselves just a few years ago. )uring the whole of the six five-year plans Russia has concentrated on the development f heavy industry at the expense of consumer goods and what we would call the ordinary eeds of a civilized society. The result is that even if one can afford the prices asked for oods in the shops they are in such short supply that persistent queueing is the only way to get them. As a result Soviet Russia has her speculator or contact man in such th as housing materials, living accommodation and motor-car spares, and at the o end of the scale the spiv who deals in stockings and household goods. There is als natural revolt of restless spirits against the drab monotony of bad housing, absen consumer goods and nothing but hard work. As a result you have the juvenile dilinq the hooligan gangs and the Teddy Boys, some of them in Edwardian jackets drain-pipe trousers just as in England. Recent visitors to U.S.S.R. have made mu these features of modern Russia but I think they have been exaggerated out of pro tion to their importance. Human nature being what it is shortages will always pro the economy of "below the counter" with the appearance of the contact men an spiv. But I think they are less evident in Russia than they were in England a decade There is even a bright side to this, for such is the demand for learning there is a b market in books—not "curious" books or banned books but the Russian classics. works of Lenin and Stalin are printed in millions: of these there are always enough. although the pre-revolutionary classics are published in editions ranging from 15,00 100,000 there are never enough to meet the demand. *War and Peace*, Chekhov's p the works of Dickens, *The Forsyte Saga*, the plays of Bernard Shaw—there are n enough of these to go round, and only a handful of the hungry multitudes are fortu enough to secure a copy. And in the winter time the principle library in Sarato 1,000 readers a day.

### CONCLUSIONS

What final impressions are left in the mind after this brief stay in Russia?

(1) First and most important the impression of a country in many ways like Victorian England must have been. A nation, virile, industrious and confident o own destiny. When we landed in Saratov we did not touch down at a modern air with offices, control tower and long runways; we came down on what was no than an open field. The fleet of cars which took us to our hotel drove through a net of unmade or badly made roads. The city looked at first sight like a familiar set fro Wild West film and one said "This is what America must have looked like when Middle West was being opened up." Russia is in the throes of her industrial revolu and she has all the certainty the Victorians had in the virtues of human and mat progress. There is the same sense of purpose, the same confidence in the future. T is also the same strain of puritanism running right through society, the same morali so revealing in their attitudes, their newspapers and their films. There is no paradi sex, no kissing in public; you never see young people walking hand in hand in pu The photograph on the front page of the newspaper is not a film star, nor the la divorcee but some worker who has laid more bricks than his fellows or a manager has raised production from fulfilment to overfulfilment in his part of the five-year p I did not see a country preparing for war. I saw a people to whom war would b disaster: who are confident that given time by economic expansion alone they will the most powerful state in the world.

(2) Secondly I saw medicine being developed along lines which made me afraid. Western civilization the healing art has always been touched by the spirit of huma or the spirit of Christianity. In Soviet Russia I saw a science and an art which ha some ways become subservient to the requirements of the State. Healing with u pursued in pity for the less fortunate without reference to the patient's usefulnes society or the economy of the country. It is not so in Russia. Hence the emph on industrial medicine and treatment aimed at fitness for employment as early possible. Hence the absence of vital statistics, the absence of Geriatric Hospitals, neglect of mental health. Human beings do not matter as human beings. While hu safety on all European air services demands the provision and strict use of safety b on aeroplanes, in most Russian planes they are dispensed with altogether. It is illustration of the different emphasis on the importance of human life. I may be wro but the stark realism of Marxist materialism when applied to medicine makes me afra

(3) My last impression is that Soviet medicine must suffer because of its lack of exibility. The methods and teaching of Pavlov are sacrosanct. This, I feel, is not ecause the Pavlov teaching of conditioned reflexes is superior to other theories in hysiology, but because it is politically convenient to make it so. It is not its scientific ccuracy that makes it the one true faith in Russian medicine and all others spurious, is because it is politically correct and fits so well with the principles of Marxism-eninism. In the building of a Communist society the conditioned reflex is a weapon of he greatest possible power; if it can be proved to be scientifically accurate, as well as olitically convenient, all the better. One can only hope that the contact being made ith the outside world since the death of Stalin will lead Soviet medicine into broader aths and make the deviationist doctor a force within the borders of the U.S.S.R.

